# Nelson’s Northern Lancet, and American Journal of Medical Jurisprudence

**Published:** 1851-11

**Authors:** 


### NELSON'S NORTHERN LANCET, AND AMERICAN JOURNAL OF MEDICAL JURISPRUDENCE.

Our excellent and intelligent cotemporary, whose name stands at the head of this paragraph, is one of the best and cheapest periodicals in the Union. We are pleased to see the increasing patronage of the work manifested in its typographical improvement. The number for October is a specimen of mechanical art, for which the editor and proprietor, Dr. Horace Nelson deserves credit and a large subscription list, and if true merit is rewarded, our confrère will secure the latter.

*Nelson's Northern Lancet* is the only medical periodical in this coun try that is mainly devoted to subjects of Medical Jurisprudence, a department of great value to the legal, as well as to the medical profession. Dr. Nelson is a master of the subject, and his papers on it display great research and marked ability. The interesting feature in his work, to which we refer should command patronage for his periodical, and we take pleasure in this humble tribute to its merits. Though its principal object is the promulgation of sound views on medical jurisprudence, the Northern Lancet is not restricted to that department. Every branch of medical science receives the aid of the work.

The periodical, whose merits have called forth these remarks, is published monthly, at Plattsburgh, New York, at the low price of one dollar per annum. It is, as we have already said, a growing work, that has commanded confidence, and will continue to do so. B.

